# Refractive Error and Axial Length and Their Related Factors in 8-Year-Old Japanese Children: The Yamanashi Adjunct Study of the Japan Environment and Children’s Study (JECS)

**DOI:** 10.3390/jcm12185929

**Published:** 2023-09-12

**Authors:** Natsuki Okabe, Airi Takahashi, Yumi Shigemoto, Chio Kogure, Tadao Ooka, Ryoji Shinohara, Sanae Otawa, Anna Kobayashi, Sayaka Horiuchi, Megumi Kushima, Zentaro Yamagata, Kenji Kashiwagi, The Yamanashi Adjunct Study of the Japan Environment and Children's Study Group

**Affiliations:** 1 Department of Ophthalmology, School of Medicine, University of Yamanashi, Chuo 409-3898, Japan; nokabe@yamanashi.ac.jp (N.O.); chio3230@yahoo.co.jp (C.K.); 2 Department of Health Sciences, School of Medicine, University of Yamanashi, Chuo 409-3898, Japan; tohoka@yamanashi.ac.jp (T.O.);; 3 Center for Birth Cohort Studies, Interdisciplinary Graduate School of Medicine, University of Yamanashi, Chuo 409-3898, Japan; rshinohara@yamanashi.ac.jp (R.S.); aumino@yamanashi.ac.jp (A.K.);

**Keywords:** refractive error, children, axial length

## Abstract

Purpose: To investigate the distribution of visual acuity, refractive error, and axial length in 8-year-old children who participated in an additional survey in Yamanashi Prefecture of the Japan Environmental Children’s Study (hereafter referred to as JECS-Y) conducted from 2019 to 2021. Participants and Methods: Eight-year-old children who participated in the JECS-Y study were subjected to noncycloplegic measurements of refractive error and axial length. If the uncorrected visual acuity was less than 20/20, the best corrected visual acuity was evaluated in accordance with the autorefraction data. A questionnaire was administered regarding the parent’s history of eyeglass wear or contact lens use. Results: Among the 400 participating children, the rate of uncorrected visual acuity of 20/20 or better in both eyes was 70.4%. The mean equivalent spherical equivalent error for both eyes was −0.366 ± 1.016 D. The mean axial length was 23.08 ± 0.225 mm in all patients. The males showed significantly longer axial length than the females despite no differences in body height. There was a significant correlation between axial length, spherical refractive, and uncorrected visual acuity. The children of parents with a history of wearing eyeglasses or contact lenses showed a significantly more myopic equivalent refractive error than those without a history. Conclusions: This study clarified the current state of refractive error in 8-year-old children and the association of inheritance with refractive error. In addition, the axials were significantly longer in male patients.

## 1. Introduction

Myopia increases the risk of developing various ophthalmic diseases, such as glaucoma, macular atrophy, and retinal detachment [1,2]. According to a recent report, approximately one-third of the world’s population has myopia, and this number is estimated to reach approximately half by 2050 [3]. In Japan, the percentage of elementary school students with uncorrected visual acuity of less than 20/20 was 17.9% in 1979 but doubled to 34.57% in 2019. Most of these cases are due to myopia, and the increase in the number of myopic children and the worsening of myopia have become social problems. In recent years, as the prevalence of myopia has increased, large-scale studies on uncorrected visual acuity and refractive error have been conducted in various regions in children. However, most of these studies are cross-sectional.

In 2011, the Japanese Ministry of the Environment launched the Japan Environment and Children’s Study (JECS). The JECS is a prospective cohort study of approximately 100,000 children across Japan, ranging from prenatal to 13 years of age. The main objective of the study is to identify the environmental factors that affect children’s health and growth and to create a healthy environment in which children can grow with peace of mind [4]. The Yamanashi Adjunct Study of the Japan Environment and Children’s Study (JECS-Y) is a study conducted in addition to the JECS that targets 4000 participants registered in the JECS who live in Yamanashi prefecture. It is a cohort study conducted in various fields, including ophthalmology, pediatrics, obstetrics and gynecology, immunology, otorhinolaryngology, endocrinology, orthopedics, and dental surgery. As part of the JECS-Y study in the ophthalmology field, we conducted a cross-sectional survey of 8-year-old children to investigate the distribution of and correlations among uncorrected visual acuity, corrected visual acuity, refractive error, and axial length, in addition to administering a questionnaire survey to family members to investigate their genetic contribution to refractive error.

## 2. Participants and Methods

This study was approved by the Ethical Review Committee of the University of Yamanashi School of Medicine and was conducted in accordance with the Declaration of Helsinki. Written consent was obtained from all the participants and their parents.

### 2.1. Enrolled Participants and Investigated Parameters

The JECS-Y survey is conducted at four-year intervals. The registered children undergo their first survey at age 4 upon entry. However, due to the challenging nature of conducting ophthalmic examinations accurately at age 4, the focus of this current survey was on children who had reached the age of 8 since their initial entry. The children included in this survey will next be examined when they turn 12 years old. In the current study, a total of 400 children (males: 187, females: 213) who were 8 years old participated in the JECS-Y survey during 2019–2020. Ophthalmologists or optometrists examined the following parameters: uncorrected visual acuity, best corrected visual acuity (BCVA), refraction error, axial length (AL), and questionnaire results regarding the parents’ confirmed history of eyeglass use or contact lens use. Visual acuity was measured in one eye at a time using a 3 m visual acuity chart (K-3801, Inami Co., Ltd., Tokyo, Japan). If the uncorrected visual acuity was less than 20/20, the corrected visual acuity was measured with reference to the refractive values measured using an autorefractometer (AR-F, NIDEK Co., Gamagori, Japan) under physiological pupil conditions. We did not use cycloplegia in this study because the JECS-Y survey committee denied its use due to difficulty in revising the ethical protocol. AL was measured using a coherence interferometry-based optical biometer (AL-Scan, NIDEK Co., Gamagori, Japan). A precise method is followed. The subjects were seated in front of an autorefractometer, AR-F, and their gaze was fixed on the target within the autorefractometer, for the measurement of refraction. The measurements were conducted following the instructions in the machine’s manual. Measurement results with a reliability coefficient of eight or higher were deemed reliable and adopted for analysis. Refraction values were first measured for the right eye, followed by the left eye. Subsequently, the subjects were seated on a chair positioned 3 m away from a visual acuity chart, K-3801, and their unaided visual acuity was assessed. The assessment began by covering the left eye and measuring the unaided visual acuity of the right eye. Visual acuity was evaluated according to the following chart: Starting from the 20/200 line, if the answer was correct, the index was adjusted step by step. If the same visual acuity index was answered incorrectly twice or more in a row, the index was lowered by one step. The index corresponding to the visual acuity achieved after two or more consecutive correct responses was recorded as the visual acuity. Then, the left eye’s visual acuity was measured like that of the occluded right eye. In cases where the unaided visual acuity was less than 1.0, corrective lenses were used for visual acuity measurement, referring to the autorefractometer’s measurement results. Subsequently, the axial length of the eye was measured. Axial length measurements were performed in automatic measurement mode using optical measurements, AL-Scan, in accordance with the machine’s manual, and reliable data were obtained through successive measurements. In this study, it was not possible for the authors to access individual participant information because it was anonymized prior to analysis.

### 2.2. Statistical Test

The statistical software Eazy R^®^ (Ver. 1.61) was used. Student’s *t* test was employed for comparisons of continuous variables between the two groups. Analysis of variance (ANOVA) or multiple comparison tests (Tukey-Kramer method) were employed to compare multiple groups. The significance level was set at less than 5%. The logarithm of the minimum angle of resolution (logMAR) and equivalent refractive error (SE) were subjected to statistical analyses. Values are presented as the mean ± standard deviation.

## 3. Result

### 3.1. Distribution of Uncorrected Visual Acuity

**Figure 1:**
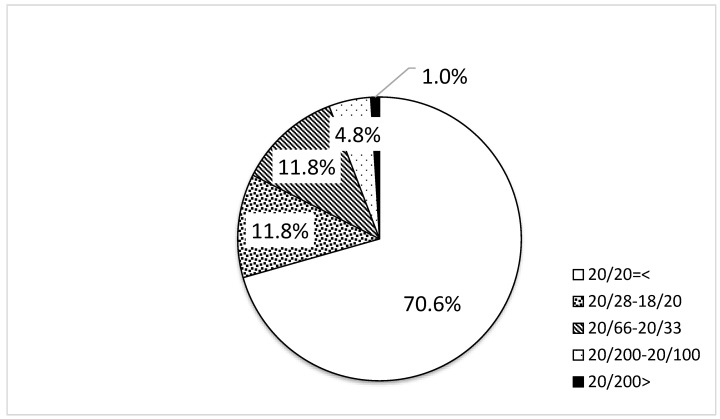
Distribution of uncorrected visual acuity.

Uncorrected visual acuity was measured in 399 of the 400 participants. Measurements of both eyes were unobtainable in one participant due to noncooperation. In one participant, visual acuity was measured for the right eye (20/20) but could not be measured for the left eye. The uncorrected visual acuity distribution is shown in Figure 1. A total of 70.6% (281 participants) had a binocular visual acuity of 20/20 or better, 11.8% (47 participants) had a visual acuity of 20/28–18/20 in either eye, 11.8% (47 participants) had 20/66–20/33, 4.8% (19 participants) had 20/200–20/100, and 1.0% (4 participants) had less than 20/200. The numbers of participants whose BCVA was better than 20/20, 20/28–18/20, 20/66–20/33, 20/200–20/100, and less than 20/200 were 15, 4, 7, 2, and 0, respectively.

There were no significant differences in the distribution of uncorrected visual acuity between the right and left eyes.

**Figure 2:**
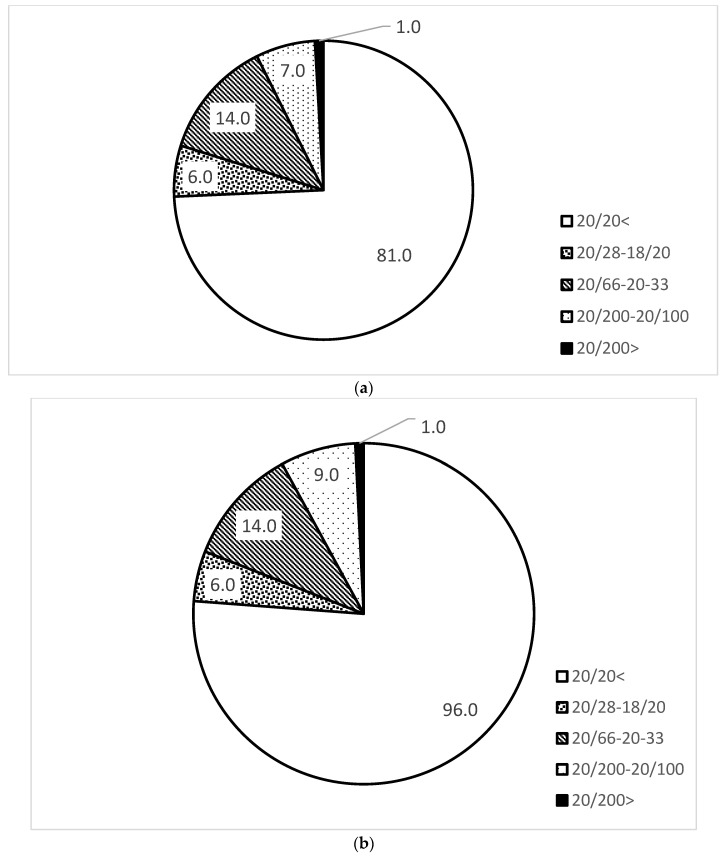
Comparison of uncorrected visual acuity between males (**a**) and females (**b**).

Comparison of the distribution of uncorrected visual acuity between males and females showed that females tended to do better, but the difference was not significant (Figure 2).

### 3.2. Distribution of Refractive Error

**Figure 3:**
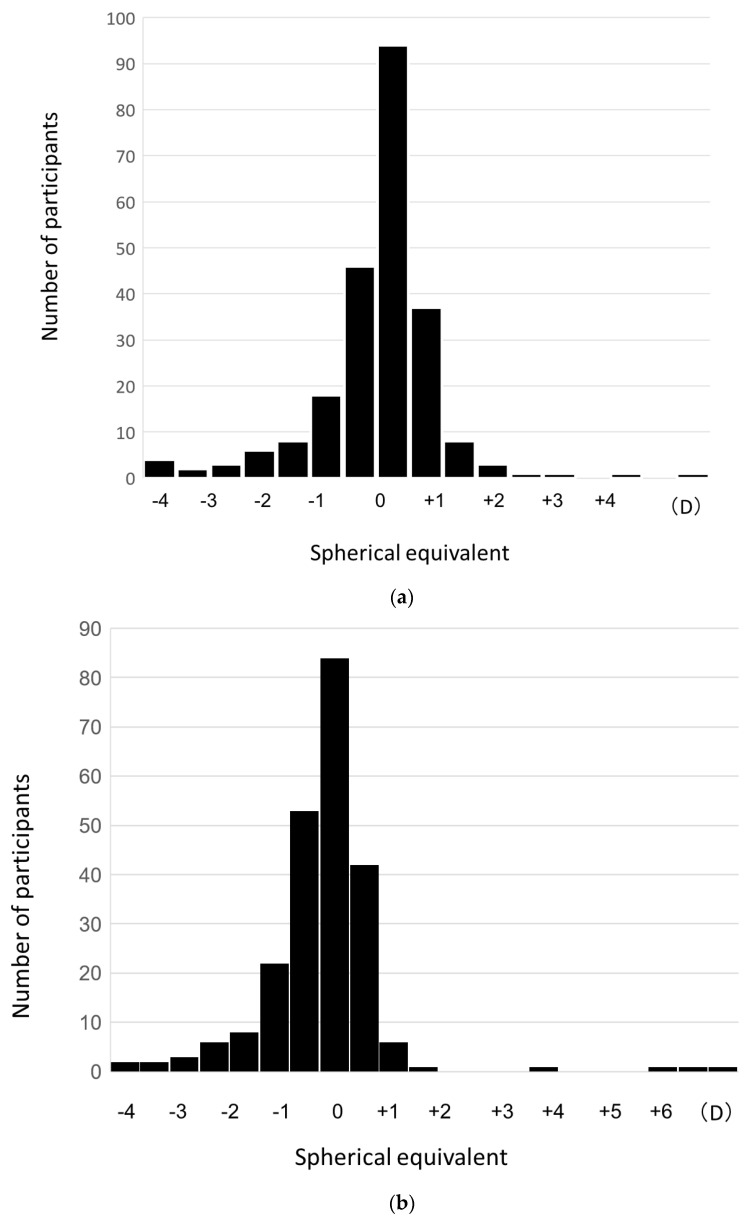
Spherical equivalent distribution of right eye (**a**) and left eye (**b**). D: diopter.

Since the autorefractometer was changed to NIDEK AR-F in November 2019, 4 months after the start of the study, we employed refractive error values measured with the NIDEC AR-F to exclude the influence of differences in the measurement instruments on the measurement results. Therefore, 233 (108 males and 125 females) participants, 58.3% of the 400 participants who were measured using this instrument, were used for this analysis. The equivalent spherical error of the right eye was −0.376 ± 0.949 D (−4.00~+4.13 D) and that of the left eye was −0.356 ± 1.073 D (−3.75~+6.25 D). There were no significant differences between the right and left eyes (Figure 3) or between males and females.

### 3.3. Distribution of Axial Length

**Figure 4:**
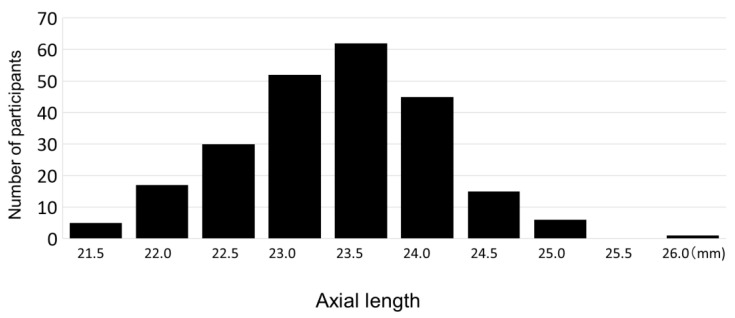
Axial length distribution.

**Figure 5:**
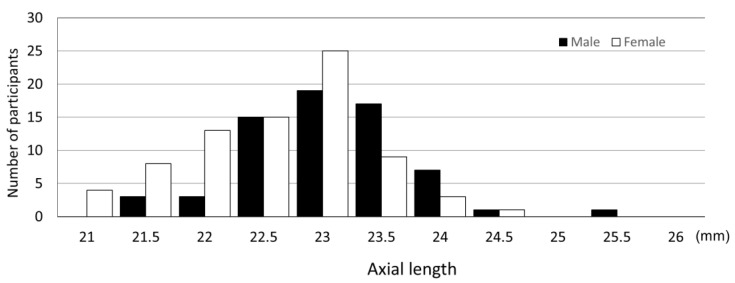
Distribution of axial length by sex.

The mean axial length of the right and left eyes was 23.08 ± 0.23 mm (range: 21.00 mm to 25.89 mm). The distribution of axial length is shown in Figure 4 and Figure 5. The mean axial length of the right eye was 23.07 ± 0.76 mm (range: 20.76 mm to 25.63 mm), showing no significant difference between the right and left eyes. Comparison of the binocular mean axial length by sex showed that the binocular mean axial length of males was significantly longer at 23.35 ± 0.71 mm (range: 21.67 mm to 25.89 mm) than that of females at 22.85 ± 0.72 mm (range: 21.00 mm to 24.74 mm) (*p* < 0.0001). In contrast, the average body heights of the males and the females were 125.06 cm and 124.31 cm, respectively, with no significant height difference (*p* = 0.29).

### 3.4. Relationship among Uncorrected Visual Acuity, Refractive Error, and Axial Length

**Figure 6:**
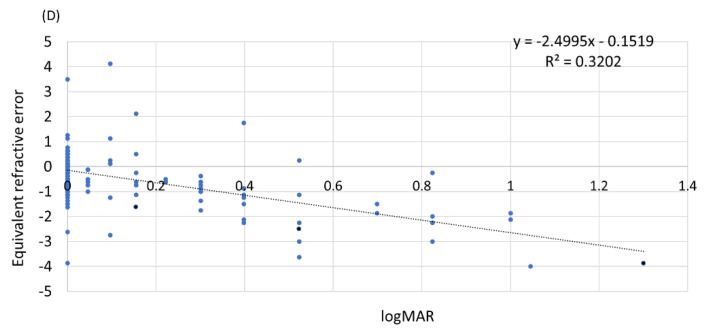
Uncorrected visual acuity (logMAR) and refractive error. D: diopter.

**Figure 7:**
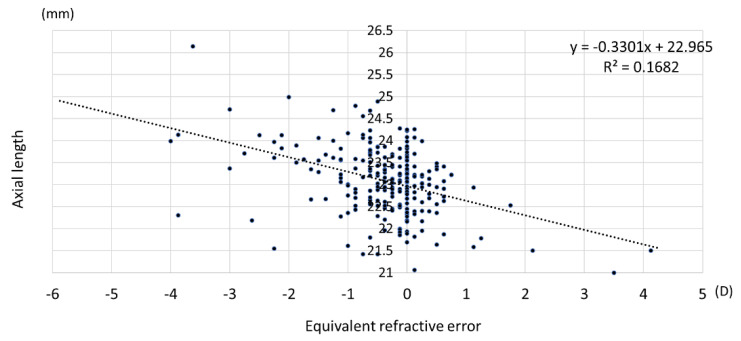
Correlation between axial length and refractive error. D: diopter.

There was a significant negative correlation between uncorrected visual acuity and equivalent spherical refractive error, with a Pearson’s correlation coefficient of −0.565 (*p* < 0.01) (Figure 6). A significant negative correlation was also found between axial length and refractive error (Figure 7), with a Pearson’s correlation coefficient of −0.319 (*p* < 0.01). This significant correlation was confirmed in males and females (*p* < 0.01).

Uncorrected visual acuity showed a significant relationship with axial length, with a Pearson’s correlation coefficient of 0.316 (*p* < 0.01).

### 3.5. Relationship between Axial Length, Refractive Error, and Body Height

**Figure 8:**
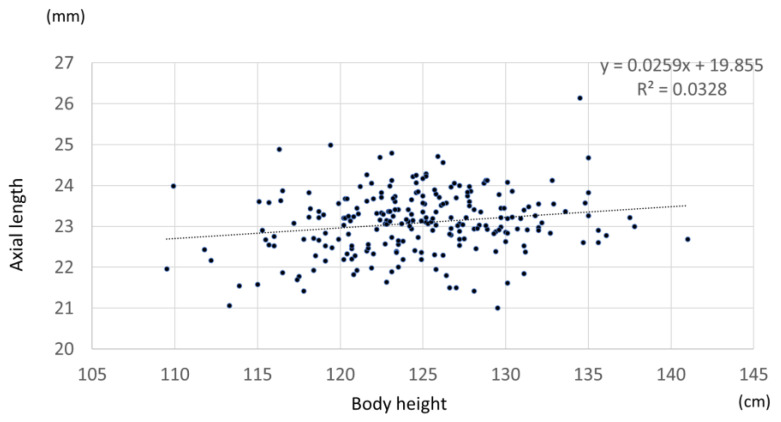
Correlation between axial length and body height.

A significant positive Pearson’s correlation coefficient of 0.187 was found between axial length and body height (*p* = 0.004) (Figure 8). A significant positive Pearson’s correlation coefficient of 0.2 was found for females (*p* = 0.025), while a positive but nonsignificant correlation was found between axial length and body height for males (*p* = 0.182). Multiple regression analysis was performed with axial length as the dependent variable and body height, sex, and spherical equivalent as the independent variables. *p* < 0.01 was obtained for all parameters. In contrast, there was no significant association between spherical equivalent and height in all cases or by sex (all cases *p* = 0.07, males *p* = 0.058, females *p* = 0.41).

### 3.6. Association between Parents’ Use of Eyeglasses or Contact Lenses and Children’s Refraction Error

**Figure 9:**
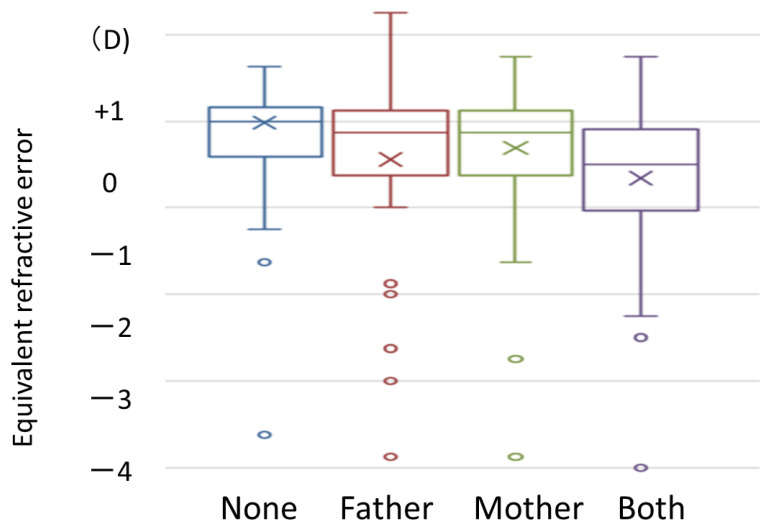
Parent’s use of eyeglasses or contacts and children’s spherical equivalent. ×: median, upper box end: upper quartile, lower box end: lower quartile, circle: outlier.

When parents were asked if they had worn eyeglasses or contacts for refractive correction by the age of 20, 75 parents had never worn eyeglasses or contacts, 73 fathers had worn eyeglasses or contacts, 96 mothers had worn eyeglasses or contacts, and a total of 117 mothers and fathers had worn eyeglasses or contacts. Myopia was the reason for wearing eyeglasses or contacts in 85.2% parents, hyperopia in 7.8%, and unknown in 7.1%. We investigated the relationship between parents’ use of eyeglasses or contacts and children’s spherical equivalent and axial length. The spherical equivalent was highest in the group whose parents had no history of use of eyeglasses or contacts and tended to be lower in the group whose one parent had a history of use and the group whose both parents had a history of use. In particular, a multiple comparison test significantly differed between the groups with no history of use and those with a history of use (*p* = 0.002, Figure 9).

### 3.7. Association between Parents’ Use of Eyeglasses or Contact Lenses and Children’s Axial Length

**Figure 10:**
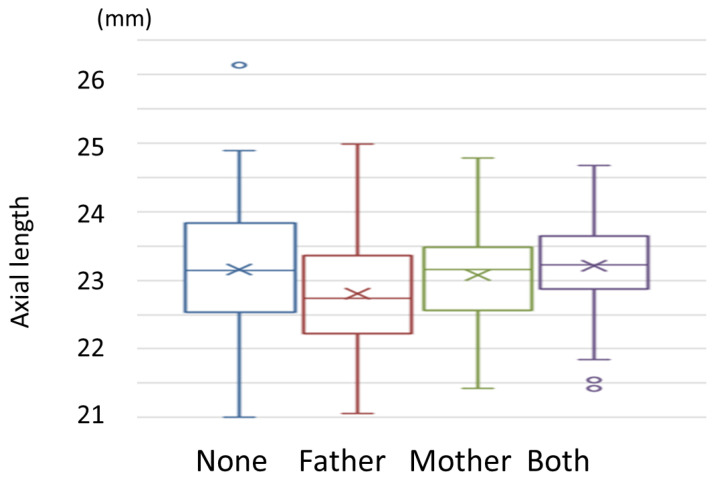
Parents’ use of eyeglasses or contacts and children’s axial length. ×: median, upper box end: upper quartile, lower box end: lower quartile, circle: outlier.

A multiple comparison test showed no significant difference among the four groups. (Figure 10) However, participants whose parents had a history of eyeglasses or contacts showed a tendency of longer AL compared with those whose parents had no history of eyeglasses or contacts.

## 4. Discussion

In this study, uncorrected visual acuity, refractive error, and axial length were investigated in 8-year-old children who participated in JECS. The results showed that approximately 30% had uncorrected visual acuity of 0.9 or less in at least one eye. The mean refractive error of both eyes was −0.682 ± 1.17 D, indicating that myopia had developed in the 8-year-olds. Although many of the results of the present study showed trends consistent with previous reports, some new findings were obtained.

A worldwide increase in myopia in school children has been reported in recent years. Similar to the present study, the results of a study with noncycloplegic data reported that 60% of children in Southeast Asia and 40% of children in North America were myopic [5]. Sato et al. studied the refractive error of 3–6-year-old children in Japan with noncycloplegic data and found a refractive error of −1.06 D. Choong YF et al. studied children in Malaysia and found that they did not use cycloplegia [6]. The refractive error in these two reports was somewhat stronger than that in the present results. A direct comparison among studies is difficult because of the involvement of race, age, and the use of cycloplegia. According to previous reports, the average spherical equivalent for children aged 6–17 years in Beijing was −1.47 D, and for children aged 6–17 years in Hebei Province, China, it was 0.07 D. Children were more likely to be affected by myopia in urban areas than in rural areas [7].

Measurement of axial length is important as an evaluation index for axial myopia because refractive error is affected by the use of cycloplegia. In the present study, axial length was measured in a larger number of participants than in previous reports. Previous reports on adults have shown that axial length correlates with height and refractive error [7]. A study conducted in children who were 9 years of age in the Netherlands reported that the axis length of the myopic group (SE ≤ −0.5 D) was 23.98 (22.75–25.37) mm, which was significantly shorter than the 22.08 (21.20–23.28) mm of the hyperopic group (SE ≥ +2.0 D) [8]. The results of the present study showed that the mean axial length was 23.08 ± 0.225 mm and that the axial length correlated with height, uncorrected visual acuity, and refractive error, which were consistent with a previous report on adults. Interestingly, the present study revealed that the axial lengths of the male children were predominantly longer than those of the female children, even after adjusting for body height, refractive error, and uncorrected visual acuity. Although it has been reported that the growth rate of the ocular axis length is faster in younger participants, to our knowledge, there are no reports on the relationship between ocular axis length and height by sex [9,10]. In Hashemi et al.’s study, regarding the comprehensive analysis of pediatric ocular structures, it was reported that among children, males have larger values in terms of axial length, anterior chamber depth, corneal curvature radius, and corneal diameter, while females have greater lens thickness [11]. In the present study, although we did not explore sex differences in anterior chamber depth, corneal curvature radius, corneal diameter, or lens thickness, we plan to investigate these ocular structures in the future. Additionally, it has been reported that there are no gender differences in axial length for infants aged 3 to 6 months [12]. It is known that girls grow faster in height than boys, but it is not clear whether there are sex differences in organ growth rates. As this study is conducted longitudinally, we also intend to examine the changes in ocular structures during growth, including potential gender differences. Currently, there is no clear evidence to explain the difference in axial length between male children and female children. Although a clear correlation between axial length and height is not evident in the current study, if there is a possibility that ocular growth precedes height growth, it suggests the potential to predict future height growth. Therefore, we plan to continue our investigations by increasing the sample size in the future.

In the present study, we examined the relationship between parents’ wearing of refractive devices and children’s refractive error and found that children were significantly more myopic in the group in which both parents wore refractive devices. The percentage of children with myopia (defined as an SE value of −1 D or less) was 13.6%: 27.7% if one parent was myopic and 58.7% if both parents were myopic [13]. Similarly, L Guo et al. examined 13-year-olds in Guangdong, China, and found that the percentage of myopia in children with two nonmyopic parents was 43.7%, while the percentage increased to 53.0% when one parent was myopic and 60.5% when both parents were myopic [14]. In the same study of 6- to 18-year-olds in Beijing, China, the results showed an association between parental myopia and child myopia: −2.33 D when both parents were myopic, −1.87 D when one parent was myopic, and −1.13 D when neither parent was myopic [14,15].

An Australian study of 12-year-olds from different ethnic backgrounds also found that 7.6% of the children were myopic if neither parent was myopic, 14.9% if one parent was myopic, and 43.6% if both parents were myopic [16]. These results are consistent with the present study. The results of the present study are evidence that refractive error and heredity have a long-lasting link. However, the percentage of children with myopia has been increasing in recent years, and environmental factors such as lifestyle are strongly considered in addition to genetic factors. Although various efforts are being made to control the progression of myopia, as shown in this study, children with genetic factors are likely to have more advanced myopia, and more attention should be given to myopia control.

Several challenges exist in this study. Because of the strong accommodative power of children, the use of cycloplegia is necessary to accurately study refractive error [15], although we did not use cycloplegia in this study. Zhu et al.’s study demonstrated that the frequency of myopia was lower when accommodative paralytic agents were used than when they were not used [17]. Another study conducted by Mimouni et al. involving 1400 individuals aged 18 to 21 years revealed that the use of accommodative paralytic agents resulted in an average hyperopic shift of 0.83 D, and the difference between using and not using these agents was more pronounced for hyperopic individuals [18]. While the use of accommodative paralytic agents is beneficial for accurately assessing refractive anomalies, it comes with various side effects and can impose burdens on investigators and subjects.

It may be better to use cycloplegia for accurate evaluation of refractive error. Since this survey is an initiative of the Japanese government and is subject to very strict regulations, the use of cycloplegia was deemed by the Steering Committee to be invasive to the participant, and its use was not permitted. However, the length of the ocular axis was measured in addition to the refractive error in this study, and we believe that the evaluation can be performed without using cycloplegia. In addition, in recent years, alternative methods have been proposed, such as measuring refractive errors without using accommodative paralytic agents and then employing artificial intelligence to estimate true accommodative disorders [19,20]. It is essential to consider and further investigate these approaches in the future. The survey of parents’ use of refractive correction devices was based on a questionnaire only and did not measure their refractive error or axial length, which raises issues of reliability. In addition, unlike epidemiological surveys, this study was conducted with voluntary participants. Therefore, the possibility of sample bias cannot be ruled out.

The present study is not considered accurate for estimating the prevalence of myopia nationwide because participants are limited to those who actively request it. However, JECS-Y conducts ophthalmologic examinations and various systemic examinations, and there is a high possibility that unknown factors related to refractive error will be revealed in the future. In addition, since the prospective study will continue for 10 more years, we will be able to observe several ocular biological and functional changes in the same children as they grow older. In addition, a detailed survey of approximately 5000 participants is being conducted, including an overall survey and genetic testing in part, which is expected to investigate refractive error and other ocular diseases and the relationship between the whole body and ocular findings. Further investigations and research will be conducted in the future. Finally, the conclusions of this article are solely the responsibility of the authors and do not represent the official views of the above government.

## Data Availability

The datasets generated during and/or analyzed during the current study are available from the corresponding author on reasonable request.
